# Reoperation-requiring postoperative intracranial haemorrhage after posterior fossa craniotomy: Retrospective case-series

**DOI:** 10.1016/j.bas.2023.102741

**Published:** 2023-12-30

**Authors:** Elise K. Kristensen, Kay Müller, Tor Ingebrigtsen, Haakon Lindekleiv, Roar Kloster, Jørgen G. Isaksen

**Affiliations:** a Department of Clinical Medicine, Faculty of Health Sciences, UiT the Arctic University of Norway, Tromsø, Norway; b Department of Neurosurgery, Ophthalmology and Otorhinolaryngology, University Hospital of North Norway, Tromsø, Norway; c Department of Radiology, University Hospital of North Norway, Tromsø, Norway

**Keywords:** Craniotomy, Craniectomy, Posterior fossa, Postoperative complications, Postoperative haemorrhage, Monitoring

## Abstract

**Introduction:**

Studies report rates of treatment-requiring postoperative intracranial haemorrhage after craniotomy around 1–2%, but do not distinguish between supratentorial and posterior fossa operations. Reports about intracranial haemorrhages’ temporal occurrence show conflicting results. Recommendations for duration of postoperative monitoring vary.

**Research question:**

To determine the rate, temporal pattern and clinical presentation of reoperation-requiring postoperative intracranial posterior fossa haemorrhage.

**Material and methods:**

This retrospective case-series identified cases operated with posterior fossa craniotomy or craniectomy between January 1, 2007 and December 31, 2021 by an electronic search in the patient administrative database, and collected data about patient- and treatment-characteristics, postoperative monitoring, and the occurrence of haemorrhagic and other serious postoperative complications.

**Results:**

We included 62 (n = 34, 55% women) cases with mean age 48 (interquartile range 50) years operated for tumours (n = 34, 55%), Chiari malformations (n = 18, 29%), ischemic stroke (n = 6, 10%) and other lesions (n = 3, 5%). One (2%) 66-year-old woman who was a daily smoker operated with decompressive craniectomy and infarct resection, developed a reoperation-requiring postoperative intracranial haemorrhage after 25.5 h. In four (6%) cases, other serious complications requiring reoperation or transfer from the post anaesthesia care unit or regular bed wards to the intensive care unit occurred after 0.5, 6, 9 and 54 h, respectively.

**Discussion and conclusion:**

Treatment-requiring postoperative intracranial haemorrhage and other serious complications after posterior fossa craniotomies occur over a wide timespan and are difficult to capture with a standardized postoperative monitoring time. This indicates that the duration of monitoring should be individualized based on assessment of risk factors.

## Highlights

• Reports about the frequency and temporal occurrence of postoperative intracranial haemorrhages in the posterior fossa show conflicting results.
• Recommendations for duration of postoperative monitoring therefore vary.
• We reviewed 62 cases operated with craniotomy or craniectomy in the posterior fossa.
• One developed a reoperation-requiring intracranial haemorrhage after 25.5 h.
• The duration of monitoring should be based on individualized risk assessment.

## 1 Introduction

Studies of patients undergoing craniotomy report rates of treatment-requiring postoperative intracranial haemorrhage around 1–2% (Kalfas and Little, 1988; Seifman et al., 2011; Wang et al., 2019; Taylor et al., 1995; Lonjaret et al., 2017; Desai et al., 2016). The studies do not distinguish between rates after supratentorial and posterior fossa operations, and they report conflicting results regarding intracranial haemorrhages’ temporal occurrence.

Recommendations for the duration of postoperative monitoring vary (Seifman et al., 2011; Valero et al., 2017). Seifman and co-workers reviewed the literature in 2011, and recommended close clinical monitoring for 6 h after all craniotomies (Seifman et al., 2011). In a more recent review, Hurtado and co-workers concluded that there is no need to systematically admit all craniotomy cases to a postoperative critical care unit (Hurtado et al., 2020). Still, they recommended that patients operated in the posterior fossa should be monitored for 24 h, due to the risk of compromising the lower cranial nerves and the increased risk of complications. However, they gave no reference to evidence supporting this.

Some neurosurgical departments, including ours, distinguish between supratentorial and posterior fossa craniotomies, and routinely monitor the latter up to 24 h (Valero et al., 2017). This is based on an assumption that an intracranial haemorrhage can cause rapidly developing respiratory failure and death, without preceding loss of consciousness (Taylor et al., 1995). We found, however, no evidence for this in the literature. Therefore, the objective of this study was to determine the rate, temporal pattern and clinical presentation of postoperative intracranial posterior fossa haemorrhage requiring reoperation within 72 h.

## 2 Material and methods

### 2.1 Study design, setting and participants

This is a retrospective study of cases operated at the University Hospital of North Norway (UNN), a tertiary regional referral centre and the only neurosurgical service for 486450 (2019) inhabitants. We included adults and children who underwent suboccipital craniotomy or craniectomy for any condition between January 1, 2007 and December 31, 2021.

All patients were routinely admitted to overnight up to 24-h postoperative monitoring, either at the post anaesthesia care unit (PACU) or the intensive care unit (ICU), depending on the availability of beds and the need for respiratory support. Both units are staffed with intensive care nurses. The ICU is also staffed with intensivists and the PACU has anaesthesiologists available on short notice. The units are equal regarding postoperative monitoring, but only the ICU has resources for continued ventilator support. No patients were monitored at the neurosurgical step-down unit or bed ward.

Postoperative computer tomography (CT) examination is not routinely done at the UNN. CT is done in cases who do not recover as expected or deteriorate after initial uneventful recovery, and in cases with new neurological deficits after the operation. When CT shows a postoperative intracranial haemorrhage, the decision to re-operate is on the surgeon's discretion.

### 2.2 Data source

Cases were identified by an electronic search identifying all cases registered with the internal operation planning code for craniectomy or craniotomy in the posterior fossa in the patient administrative system. All data were extracted from the electronic health record by reviewing the procedure note, subsequent notes made during the next 72 h and the discharge summary.

### 2.3 Variables

We extracted and registered patients’ age, sex and the condition causing the suboccipital craniotomy. Possible risk factors for postoperative intracranial haemorrhage were recorded: hypertension (yes or no), cardiovascular disease (yes or no), diabetes (yes or no), use of anticoagulant and/or antiplatelet therapy (yes or no) and tobacco use (cigarette smoking and/or snuff) (yes, former >6 months or no).

The date, time and scheduling of the operation (scheduled or emergency) and the unit for postoperative monitoring (PACU or ICU) were also registered.

The main outcome was treatment-requiring postoperative intracranial haemorrhage diagnosed by CT occurring within 72 h (yes or no). Secondary outcomes were occurrence of other adverse events (yes or no) within 72 h: Extracranial haemorrhagic complications, declining level of consciousness and/or increasing intracranial pressure not related to postoperative intracranial haemorrhage, respiratory failure requiring ventilator treatment, transfer from the PACU to the ICU and death by any cause. We also recorded the time point (hours after the operation) for onset of these events.

## 3 Statistical analysis

We present category data as counts with proportions (percentages) and continuous data as medians with interquartile ranges (IQR).

### 3.1 Ethics

The data protection officer at the UNN approved the project as quality improvement not requiring informed consent or ethics committee application (project ID: 2832).

## 4 Results

The electronic search identified 63 cases operated with suboccipital craniotomy or craniectomy. We excluded one case operated with ligation of extracranial feeders to an AVM (and not with craniotomy).

Table 1 shows that the 62 included cases were 34 women (55%) and 28 (45%) men with a median age of 48 (range 1–82) years. Tumours (n = 34, 55%) and Chiari malformations (n = 18, 29%) were the most frequent conditions. Excision or resection of a tumour or another lesion (n = 39, 63%) and occipitocervical decompression (n = 20, 32%) were the most frequent surgical treatments. Most of the operations (n = 57, 92%) were scheduled. Until 2019, most cases (n = 33/47, 70%) were transferred to the PACU for postoperative monitoring. In 2020 and 2021, all cases (n = 15) were monitored in the ICU during the COVID-19 pandemic.

**Table 1:** Baseline and treatment characteristics of 62 cases undergoing posterior fossa craniotomy.

Demographics	
Age, years, median (IQR)	48 (50)
Sex, female, n (%)	34 (55)
Condition in the posterior fossa	
Chiari malformation, n (%)	18 (29)
Primary tumour, n (%)	18 (29)
Metastasis, n (%)	16 (26)
Ischemic stroke, n (%)	6 (10)
Other cerebrovascular disease, n (%)	3 (5)
Other, n (%)	1 (2)
Risk factors for postoperative ICH	
Anticoagulant therapy, n (%)	9 (15)
Hypertension, n (%)	18 (29)
Cardiovascular disease, n (%)	13 (21)
Diabetes, n (%)	4 (6)
Tobacco use	
Yes, n (%)	21 (34)
Former, n (%)	8 (13)
No, n (%)	15 (24)
Missing, n (%)	18 (29)
Surgical treatment	
Excision or resection of tumour or other lesion, n (%)	39 (63)
Occipitocervical decompression, n (%)	20 (32)
Other, n (%)	3 (5)
Timing of the operation	
Scheduled, n (%)	57 (92)
Emergency, n (%)	5 (8)
Unit for postoperative monitoring	
ICU, n (%)	33 (53)
PACU, n (%)	29 (47)

A reoperation-requiring postoperative intracranial haemorrhage in the posterior fossa occurred in one (2%) case, 25.5 h after the index operation. The patient was a 66-year-old woman who was a daily smoker and had a sinoatrial block causing bradycardia. She was admitted with an expansive cerebellar infarction causing acute hydrocephalus and operated with decompressive craniectomy and resection. After an uneventful course for 24 h, she deteriorated rapidly after transfer to the bed ward, with the level of consciousness declining to Glasgow Coma Scale score 6, gauze deviation and dilation of the pupils. She was reoperated with evacuation of the haematoma and the subsequent course was uneventful.

In four (6%) cases, other treatment-requiring complications occurred within 72 h. The first case was a large extracranial wound haemorrhage occurring at the PACU after 0.5 h in a 77-year-old male previous smoker who used antiplatelet medication. It required urgent wound revision. The second was a 48-year-old male with aspiration-related pneumonia requiring transfer from the PACU to the ICU after 6 h for continued airway management. The third was a 68-year-old woman with hypertension and cardiovascular disease who developed sinus vein thrombosis and hydrocephalus and was re-operated with external ventricular drainage after 9 h. The fourth case was a 71-year-old man who underwent a decompressive craniectomy for a cerebellar infarction. After an initially uneventful postoperative course, he suffered sudden respiratory arrest at the bed ward after 54 h, and subsequently died from global ischemic brain damage. No clear cause could be established. Autopsy was not done.

## 5 Discussion

### 5.1 Key results

The main finding in this retrospective case-series is that one (2%) case developed a reoperation-requiring postoperative intracranial haemorrhage 25.5 h after the index operation. Another four (6%) cases developed other serious treatment-requiring complications after 0.5, 6, 9 and 54 h, respectively. Two were diagnosed at the PACU within 6 h and one at the ICU after 9 h. The two most serious complications (the intracranial haemorrhage and a case of respiratory arrest) occurred at the bed ward after more than 24 h. Accordingly, the postoperative intracranial haemorrhage that required reoperation would not have been captured by the routine 6-h monitoring recommended for all craniotomies in the review by Seifman and co-workers (Seifman et al., 2011), and neither was it captured by our institution's up to 24 h monitoring routine.

### 5.2 Interpretation

The rates of treatment-requiring postoperative intracranial haemorrhage and other serious treatment-requiring complications in the present case-series are comparable with previous studies of patients undergoing craniotomy (Kalfas and Little, 1988; Seifman et al., 2011; Wang et al., 2019; Taylor et al., 1995; Lonjaret et al., 2017; Desai et al., 2016). In 1995, Taylor and co-workers reported a postoperative intracranial haemorrhage rate of 2.2% among 2305 patients undergoing various supra- and infratentorial intracranial operations (Taylor et al., 1995). More recently, Wang and co-workers reported a rate of 1.8% in a series of 2259 cases operated for intracranial tumours, with no difference between supratentorial and posterior fossa operations (Wang et al., 2019).

Few have reported the temporal occurrence of postoperative intracranial haemorrhage after operations in the posterior fossa (Taylor et al., 1995; Desai et al., 2016). Taylor and co-workers found that 44/50 (88%) of the haemorrhages occurred within 6 h, none between six and 24 h and 6/50 (12%) after more than 24 h. They did not specify the location of the craniotomies, but nevertheless recommended prolonged monitoring (beyond 6 h) of cases operated in the posterior fossa, assuming a risk of hydrocephalus and lower cranial nerve palsies with an associated threat to airway and respiratory response (Taylor et al., 1995). This assumption has been frequently cited and seems to be the main source for recommendations that patients operated in the posterior fossa should be monitored for 24 h. Hurtado and co-workers recently reviewed the literature on postoperative circuits in patients undergoing elective craniotomy and suggested a decision algorithm (Hurtado et al., 2020). This algorithm recommends 24 h monitoring of cases operated in the posterior fossa, based on the same reasoning. Interestingly, in our study, two of the five serious complications occurred within 6 h, while two of the other three, including the treatment-requiring intracranial haemorrhage, occurred after more than 24 h. Accordingly, the optimal duration of routine postoperative monitoring after posterior fossa surgery remains unclear.

The cases who developed haemorrhagic complications in the present series harboured established risk factors such as smoking, vascular disease and use of antiplatelet therapy (Seifman et al., 2011). The case who developed a postoperative intracranial haemorrhage underwent resection of a cerebellar infarction. Emergency operation and a large tissue surface after tumour or infarct resection could be risk-factors, but the small sample size in this study precludes us from drawing conclusions. The decision algorithm suggested by Hurtado and co-workers recommend customized duration of the postoperative monitoring, based on an assessment of individual patients’ risk profile (Hurtado et al., 2020). However, from the literature, the evidence for inclusion of posterior fossa operation as a risk factor in such assessments is weak. The suggested algorithm is complex, and it could be argued that standardized postoperative circuits reduce the risk for assessment errors. Based on a discretionary assessment of the present study and the literature, we have not changed our established routine of overnight and up to 24 h monitoring after posterior fossa craniotomy.

### 5.3 Limitations

The present study is small and retrospective. We cannot preclude that some posterior fossa operations were misclassified as supratentorial craniotomies in our patient administrative system. This limits both internal and external validity.

A power calculation assuming the incidence rate (2 %) of postoperative intracranial haemorrhage in the present study and 80 % power with a one-sided significance level of 0.05 indicates that a prospective randomized study must include 3652 cases to demonstrate non-inferiority between the two monitoring strategies. A propensity-score matched comparative effectiveness study of cases operated at hospitals with different monitoring routines could be a more feasible study design.

## 6 Conclusions

In the present case series of 62 cases operated in the posterior fossa, reoperation-requiring postoperative intracranial haemorrhage occurred in one (2%) case after 25.5 h. The duration of postoperative monitoring after such operations should be individualized based on assessment of risk factors.

## Funding

This research did not receive any specific grant from funding agencies in the public, commercial, or not-for-profit sectors.

## Declaration of competing interest

The authors declare that they have no known competing financial interests or personal relationships that could have appeared to influence the work reported in this paper.
